# Investigations of the Influences of Processing Conditions on the Properties of Spray Dried Chitosan-Tripolyphosphate Particles loaded with Theophylline

**DOI:** 10.1038/s41598-020-58184-3

**Published:** 2020-01-24

**Authors:** Yang Wei, Yu-Hung Huang, Kuo-Chung Cheng, Yu-Lin Song

**Affiliations:** 1 Department of Chemical Engineering and Biotechnology, National Taipei University of Technology, Taipei, 106 Taiwan; 2 Department of Bioinformatics and Medical Engineering, Asia University, Taichung, 413 Taiwan; 3 Department of Computer Science and Information Engineering, Asia University, Taichung, 413 Taiwan

**Keywords:** Mechanical engineering, Biomedical engineering, Drug development

## Abstract

The preparation of chitosan-tripolyphosphate (chitosan-TPP) particles by the spray drying had been reported word widely for a sustained release of drugs to prevent rapid drug metabolism. Although the spray drying is a straightforward procedure turning a liquid feed into a well-defined dry powder, seldom research works were focusing on how the processing parameters and liquid feeding constitutions of spray drying system might affect the properties of spray-dried chitosan particles loaded with drugs, such as the particle size and morphologies, which would be very important to drug encapsulation and dissolution of the drug delivery design. This study thus prepared the chitosan particles with theophylline (TH) loaded as a model drug and TPP as cross-linker at various spray drying conditions. Our results indicate the diameter of the TH/chitosan-TPP particles made by customized spray drying apparatus spans from 424 to 497 nm with a geometric standard deviation of less than 2. The corresponding release of TH was tunable by the chitosan-TPP matrix density under the selected spray drying temperature and the carrying air flow rate. These results suggest an indeed need for optimized spray drying processing conditions to make the ideal spray-dried TH/chitosan-TPP particles for the desired drug delivery.

## Introduction

The delivery of the drug-loaded system to the site of the interest has been developed to minimize the loss and the degradation of the drug from the surrounding medium or processing condition, attaining the desired therapeutic concentration of the drug at the selected site^1^.

Recently, a biopolymer has been increasingly applied in the design of controlled drug release due to its compatibility as the drug carrier material^2^. Among which, chitosan has received lots of attention due to its biocompatibility, low toxicity, and biodegradability^3,4^. The technique employed to prepare chitosan drug carriers include nanosphere encapsulation, ionotropic gelation, coacervation, emulsification/solvent evaporation, and spray drying^5,6^. The strategy of spray drying was usually selected because it can be easily used in an aqueous or organic solvent with a rapid one-step production process, which converts a liquid feed (precursor solution) into solid particles by atomization in a continuous processing operation^7^. The active ingredient was entrapped in a biopolymer carrier under a hot drying medium. Various applications of spray-dried chitosan microspheres in drug delivery have been published^8–11^.

However, chitosan is a polysaccharide material with high viscosity which makes difficulties in the optimized control over the initial formulation (precursor solution) and operation conditions of a spray dryer^11^, which may provide a broad distribution of particle size leading to a reduced delivery effectiveness of the drugs encapsulated and causing a crucial problem in drug delivery design for pharmaceutical applications^12,13^. For example, particle sizes of spray-dried chitosan particles had been reported to be determined not only by the spray drying process parameters but also by the precursor concentration of feed solutions^7^. Furthermore, chitosan particles made by spray drying without cross-linker addition was known to be easily aggregated due to its swelling and dissolution properties^7,14^. The addition of a crosslinking agent in a precursor solution is then another required parameter that needs to be controlled for a better drug delivery design. Since commonly used cross-linking agents such as glutaraldehyde are toxic^15^, it is not allowed in pharmaceutical applications. Tripolyphosphate (TPP) anions were then used as a cross-linking agent of chitosan in this study due to its low toxic nature^7,14,16^. TPP can interact with cationic chitosan through electrostatic forces^17^, with the free amino groups in chitosan carrying positive charges for crosslinking reaction with negatively charged anions from TPP.

Theophylline (TH) has been well established orally, intravenously delivered, or inhaled as an anti-inflammatory drug^18^ for asthma^19,20^. However, recent studies indicated that the dose range of TH which optimize between efficacy and toxicity is narrow (i.e., therapeutic index of TH is between 10 to 20 μg/mL)^19,21^ and the short half-life (i.e., six h) of TH may lead to poor patient compliance due to frequent administration of drugs to avoid large fluctuations of TH concentration in plasma^22^. A sustained TH release carrier such as the TH encapsulated chitosan particles by spray drying process was required for a controlled release in the drug-delivery system to surmount these drawbacks^19,23^. However, majority of these works were focusing mainly on the properties of drug-delivery system but miss the chance to study how the spray drying process conditions may affect the desired properties of TH/Chitosan-TPP particles, from which the aim of this study investigates the correlations between the processing parameters (e.g., airflow rate from atomization and inlet temperature in heat column), properties of the liquid feed (e.g., various concentrations of chitosan and TPP in precursor solution) and the corresponding characteristics of chitosan particles containing TH as model drug and TPP as crosslinker. The particle sizes, surface morphologies and drug dissolution profiles of TH will be reported and discussed under the proposed spray drying conditions with a home-made spray dryer for the sustained release of TH in our TH/Chitosan-TPP delivery system.

## Results

The particle size, shape and surface morphology of spray-dried TH/Chitosan-TPP particles were reported under various precursor concentrations and processing parameters (i.e., the inlet flow rate of air and inlet temperature (Tc)), with the correlations between the spray drying conditions and the general properties of spray-dried TH/Chitosan-TPP drug delivery systems investigated.

### Physical properties of spray dried TH/Chitosan-TPP particles

In this study, SEM pictures of spray-dried TH/Chitosan-TPP were taken, and the corresponding particle size distributions were measured under various operating conditions, with particle morphology and mean size of each case determined by SEM photos with the assistance of software and the accuracy could be down to the resolution limit of the microscope applied (e.g., <10 nm)^24^. SEM yields the most direct information on size, size distribution, and shape of the particles compared to another commonly used technique for particle size measurement, dynamic light scattering (DLS). But it is important to note that the particle size measured is smaller in the SEM micrographs than on DLS results because the latter one measures the hydrodynamic diameter^25^. In the following sections, the diversity of the particle size (σ_g_) is described by the geometric standard deviation (G.S.D)^26^ according to Eq. (2) in section 4.5, with G.S.D approximately equal to 1.00 when the uniform and monodispersed particle population was observed^27^ while the G.S.D value of a poly-dispersed particles population is usually more than 1.50^28^.

#### Effect of chitosan precursor concentrations

**Figure 1:**
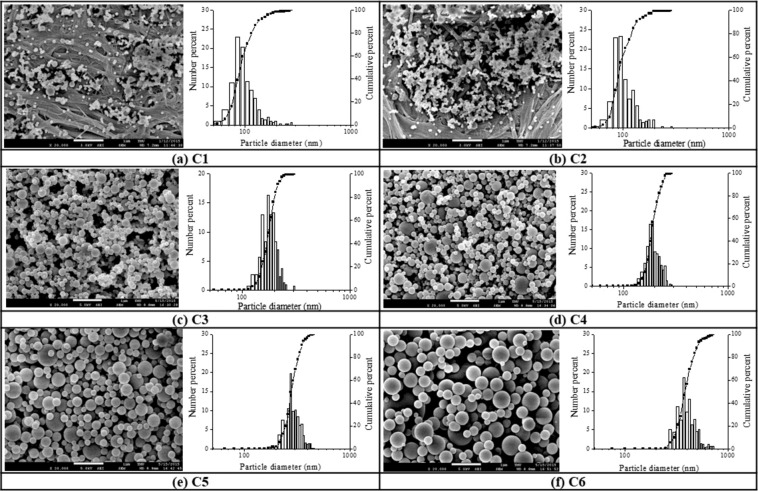
SEM images and corresponding size distributions of the chitosan particles prepared from various precursor concentrations (Table 1). (**a**) C1 (5 × 10^−5^ w/v%), (**b**) C2 (5 × 10^−4^ w/v%), (**c**) C3 (5 × 10^−3^ w/v%), (**d**) C4 (0.01 w/v%), (**e**) C5 (0.05 w/v%), and (**f**) C6 (0. 1 w/v%), at a fixed airflow rate of 0.075 L/min and a constant inlet temperature of 130 °C. (scale bar = 1 µm).

The first formulation variables under the investigation in the liquid feed are the chitosan precursor concentrations in acetic acid 1% (v/v) with the absence of TH and TPP. The viscosity of 1 wt% chitosan solution in 1% v/v acetic acid was 9.5 1.3 (cP) with n = 6, with the molecular weights determined by the method of Hirai was 37000 Da based on viscosity^29^. The diameter and size distribution of the spray-dried chitosan particles made at a constant airflow rate of ±0.075 L/min and inlet temperature (Tc) of 130 °C were shown in Fig. 1. The chitosan concentrations in the formulation were increased from 5 × 10^−5^ to 0.1 w/v% with the corresponding sample codes designated from C1 to C6. As shown in Table 1, increasing the chitosan concentration of the precursor solution (i.e., C1 to C6) caused the increased particle size with averaged diameters rising from 98 up to 415 nm of the spray-dried chitosan particles, which is in agreement with the morphological observations in Fig. 1, showing smaller particles with doughnut-like deflated balloon or hollow hemispheres shapes when chitosan precursor concentration is lower; while larger and smoother particle surfaces were obtained at higher chitosan precursor concentration.

**Table 1:** Mean size of chitosan particles prepared under various precursor concentrations in a fixed flow rate of air: 0.075 L/min and inlet temperature (Tc): 130 °C.

Sample code	Chitosan (%,w/v)	TH (%,w/v)	TPP (% w/v)	Mean Size (nm)	G.S.D (σ_g_)
C1	5 × 10^−5^	0	0	98	1.28
C2	5 × 10^−4^			101	1.27
C3	5 × 10^−3^			174	1.12
C4	0.01			185	1.17
C5	0.05			280	1.17
C6	0.1			415	1.19

σg describes the size dispersion of chitosan particles relative to their geometric mean values. Averaged standard deviation of mean size from overall data (not shown) is 20 (nm) with N > 300.

#### Effect of processing parameters (drying temperature and air feed flow rate)

**Figure 2:**
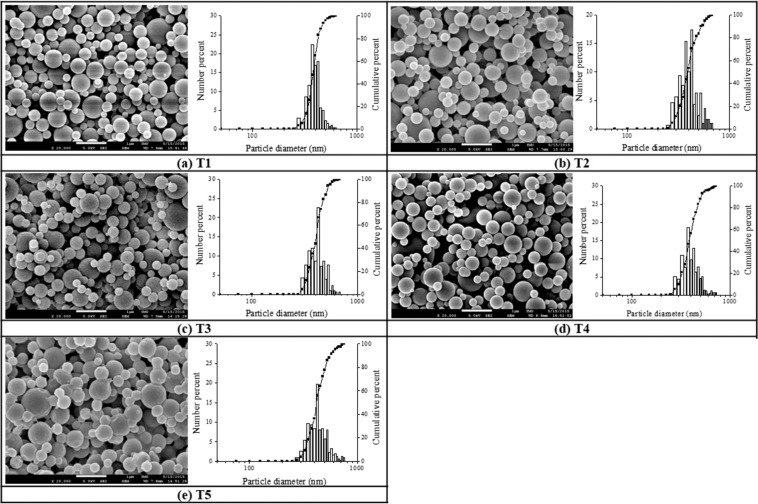
SEM images and corresponding size distributions of the chitosan particles prepared from several controlled temperatures. (**a**) T1, 70 °C, (**b**) T2, 90 °C, (**c**) T3, 110 °C, (**d**) T4, 130 °C, and (**e**) T5, 150 °C) at a fixed chitosan concentration 0.1 w/v% and the flow rate of air 0.075 L/min. (scale bar = 1 µm).

**Figure 3:**
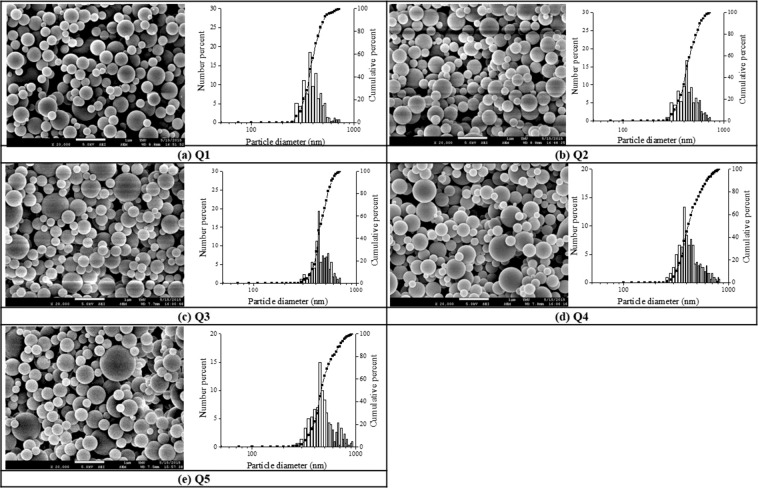
SEM images of the chitosan particles prepared from several flow rates of air. (**a**) Q1, 0.075 L/min, (**b**) Q2, 0.378 L/min, (**c**) Q3, 0.681 L/min, (**d**) Q4, 0.983 L/min, and (**e**) Q5, 1.589 L/min at a fixed chitosan concentration of 0.1 w/v% and the controlled inlet temperature of 130 °C. (scale bar = 1 µm).

The physical properties of the spray-dried chitosan particles including the particle size and size distributions of chitosan were investigated at different spray drying operating conditions such as air inlet temperature and air feed flow rate from atomization as displayed in Figs. 2 and 3, with manipulated process parameters listed in Table 2 for varying the inlet temperature (Tc) from 70 to 150 (°C) and the airflow rate from 0.075 to 1.589 (L/min.), with corresponding sample codes of T1 to T5 and Q1 to Q5, respectively.

**Table 2:** Mean size of chitosan particles prepared under various processing parameters, including the flow rate of air (i.e., Q1 to Q5) and the inlet drying temperature (Tc) (i.e., T1 to T5) at a chitosan precursor concentration of 0.1 w/v%. σg describes the size dispersion of chitosan particles relative to their geometric mean values.

Sample code	Air Flow (L/min.)	T_c_ (°C)	Mean Size (nm)	G.S.D (σ_g_)
Q1	0.075	130	415	1.19
Q2	0.378		420	1.25
Q3	0.681		447	1.20
Q4	0.987		463	1.30
Q5	1.589		487	1.28
T1	0.075	70	383	1.11
T2		90	395	1.19
T3		110	409	1.15
T4		130	415	1.19
T5		150	431	1.24

Averaged standard deviation of mean size from overall data (not shown) is 44 and 40 (nm) for sample Q and sample T, respectively, with N > 300.

As shown in Table 2, when the concentration of chitosan and the airflow rate was fixed at 0.1 w/v% and 0.075 L/min, respectively, the size of chitosan particles from spray dryer were increased by 48 nm in terms of particles’ mean diameters as the air inlet temperature was changed from 70 °C to 150 °C with sample codes of T1 to T5. Similar trend was also observed in Table 2 when the mean particle diameters of chitosan particles from spray dryer were increased by 72 nm with the increased air feed flow rate from 0.075 to 1.589 L/min (i.e., sample codes of Q1 to Q5) at the fixed chitosan precursor concentration and the air inlet temperature of 0.1 w/v% and 130 °C, respectively, from which the feed flow rate shows stronger impact when compared to inlet temperature on controlled size of the finished chitosan particles in our spray dryer.

#### Effect of crosslinker contents

**Figure 4:**
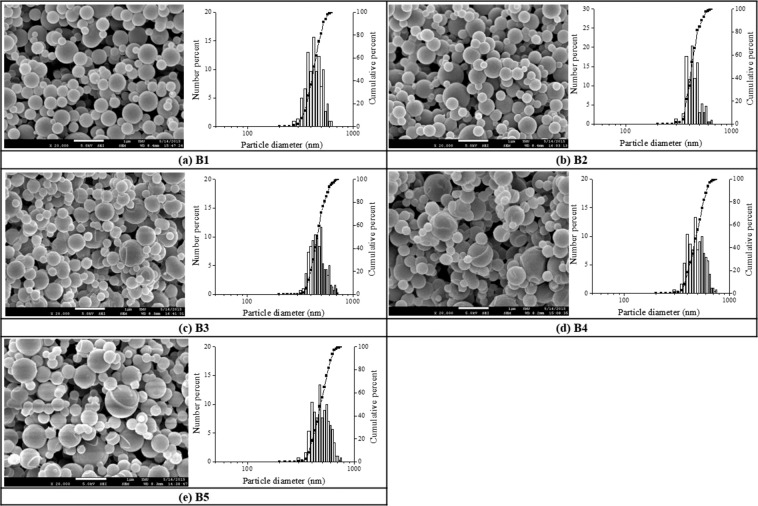
SEM images of the drug-loaded TH/Chitosan-TPP particles prepared from several TPP/Chitosan mass ratios. (**a**) B1, 0.06, (**b**) B2, 0.08, (**c**) B3, 0.1, (**d**) B4, 0.2 and (**e**) B5, 0.4 w/v% at a fixed chitosan concentration of 0.1 w/v%, the airflow rate of 0.075 L/min and the inlet temperature of 70 °C. (scale bar = 1 µm).

The effect of various TPP cross-linker amounts on particle sizes of spray-dried TH/Chitosan-TPP particles was investigated at constant formulation concentrations of TH and chitosan, 0.02 w/v% and 0.1 w/v% respectively. The selected operating conditions of constant inlet temperature and airflow rate were 70 °C and 0.075 L/min, respectively, with various amounts of TPP applied designated as B1 to B5 shown in Table 3. The lower inlet temperature selected in this study for drug-related tests was due to the concerns of none-stabilized drug structures at a higher temperature (e.g., protein drugs)^30^. As shown in Table 3, when the weight to volume percentage of cross-linker in chitosan precursor solution was increased from 0.006 w/v% to 0.04 w/v%, the mean particle diameter of the TH/Chitosan-TPP particles increased from 424 nm to 497 nm, probably due to the higher TPP/chitosan ratio leading to the excess amount of TPP depositions on top of the particle surface, causing a relatively rough surface in morphological aspect in Fig. 4.

**Table 3:** The size of TH/Chitosan-TPP particles prepared under the various amount of TPP was statically analyzed.

Sample code	Chitosan (%,w/v)	TH (%,w/v)	TPP (% w/v)	Mean Size (nm)	G.S.D (σ_g_)
B1	0.10	0.02	0.006	424	1.18
B2			0.008	430	1.11
B3			0.01	464	1.18
B4			0.02	487	1.17
B5			0.04	497	1.18

Fixed chitosan and TH precursor concentration was designated at 0.1 w/v% and 0.02 w/v%, respectively. 0.075 L/min and 70 °C were picked up as the flow rate of air and inlet temperature (Tc), respectively. σg describes the size dispersion of TH/Chitosan-TPP particles relative to their geometric mean values. Averaged standard deviation of mean size from overall data (not shown) is 45 (nm) with N > 300.

**Figure 5:**
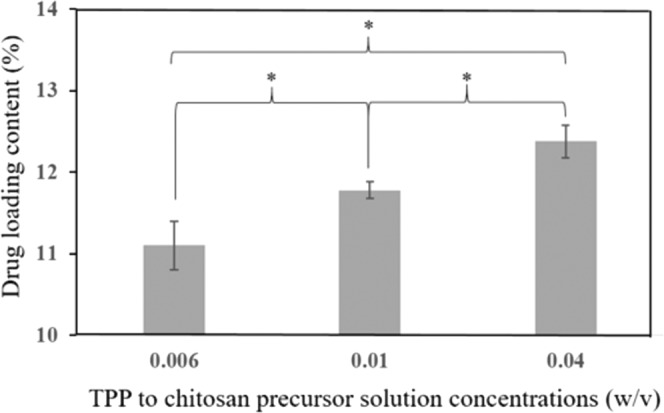
The drug encapsulation efficiency of TH with various amounts of TPP in chitosan precursor solution at constant formulation concentrations of TH and chitosan of 0.02 w/v% and 0.1 w/v% respectively, at 70 °C of inlet temperature and 0.075 L/min of airflow rate. (averaged values ± standard deviation with n = 6) (*P < 0.05).

Various amounts of TPP in chitosan precursor solution were also tested on the drug encapsulation efficiency (i.e., drug loading content) of TH at constant formulation concentrations of TH and chitosan of 0.02 w/v% and 0.1 w/v% respectively, at 70 °C of inlet temperature and 0.075 L/min of airflow rate. As shown in Fig. 5. our results demonstrated that the encapsulation efficiency of TH in our chitosan-TPP particles was affected by the amount of TPP added, with the increasing ratio of TPP to chitosan precursor solution concentrations increased the drug loading content. Such an increasing trend could be attributed to the increased free volume spaces within the polymer matrix as a result of the increased size of TH/Chitosan-TPP particles obtained from higher TPP to chitosan ratio, thereby increasing their encapsulation efficiencies.

### Drug release study

Regarding the dissolution apparatus applied in our study, instead of bringing the samples to a UV spectrometer, we have fiber optics dissolution system for this study to bring the UV spectrometer to the sample solutions with a real-time drug release level determined *in-situ* without sample removal, which has been shown to effectively record the dissolution profile of drugs in a much more reliable manner^31,32^.

**Figure 6:**
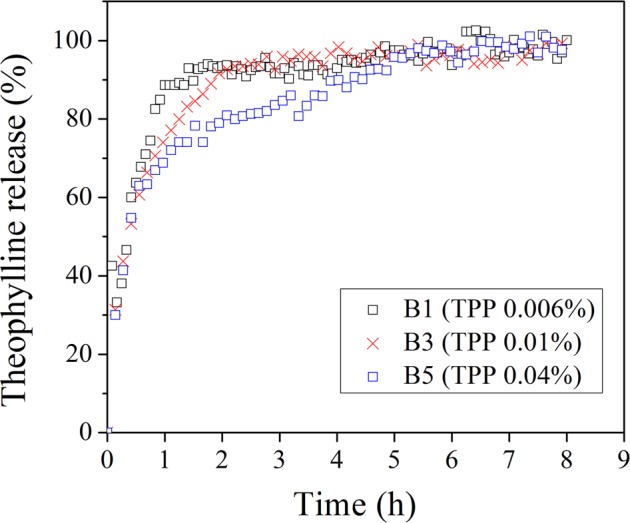
The influence of different TPP percentages (w/v) in the precursor solution on the TH releasing experiments at the fixed chitosan concentration of 0.1 w/v%, TH content of 0.02 w/v%, the flow rate of air 0.075 L/min, and the inlet temperature of 70 °C. Within the first two hours, values represent averaged drug-releasing ratios ± SD (not shown) showing significant difference with p < 0.05 for B1 versus B3 while between the time frame of 2 to 5 hours, the significant difference regarding the drug-releasing ratios was observed with p < 0.05 for B5 versus B1 and B3, n = 6.

As the selected operating conditions applied at constant inlet temperature and airflow rate of 70 °C and 0.075 L/min respectively, the releasing profile of TH from TH/Chitosan-TPP particles was influenced by the amount of TPP added, with the concentration of TPP increased from 0.006 w/v% to 0.04 w/v%, the corresponding releasing rate of TH was decreased (i.e., Fig. 6).

After the initial burst within one hour for all of the cases, the swelling and the diffusion of the target drug from the spray-dried TH/Chitosan-TPP particles continues a quick release for two hours (with 90% of initially loaded TH released within 2 hours.) when TPP content was lower than 0.04% (w/v) in the precursor solution (i.e., B1 and B3 in Fig. 6.) while the B5 curve in the same figure (i.e., TPP content in the precursor solution = 0.04% (w/v)) shows the slowest drug releasing rate, with 90% of initially loaded TH issued within 5 hours. For comparison’s sake, the dissolution profiles of the commercial capsules with prolonged release of TH micro granules in the medium of pH 7.5 was reported to have a rapid releasing rate, with nearly 100% of the initially loaded TH released within one hour^33^.

## Discussions

To investigate how the processing parameters and liquid feeding constitutions of spray drying system might affect the properties of spray-dried chitosan particles loaded with TH, the particle sizes, surface morphologies and drug dissolution profiles of TH was reported and discussed in this study under the proposed spray drying conditions with a home-made spray dryer for the sustained release of TH in our TH/Chitosan-TPP delivery system.

The influence of the chitosan precursor concentration on spray-dried particle size might be due to the volume occupied by the biopolymer in a solution^34^, with the increased chitosan concentrations of the precursor solution (i.e., C1 to C6) increasing the spray-dried particle size. For example, higher occupancy and intrinsic viscosity from higher chitosan concentration caused the entanglement of biopolymer coils with each other and the subsequent aggregates such as the filled spherical grains making bigger spray-dried particles in Fig. 1(c)–(f). When chitosan concentration is lower, biopolymers are free to move, and the coils do not entangle with each other which behaves a better solubility and decreased viscosity resulting in the increased buckling times^35^, from which a cavity and hollow grain was formed much more frequently as shown in Fig. 1(a),(b) with much smaller size of non-spherical structures obtained. Therefore, spray-dried particles with required size and morphology might be prepared by adjusting the chitosan concentration in the precursor solution, with most uniformed and smooth particle surfaces obtained at a chitosan precursor concentration of 0.1 w/v% in our spray drying system.

The reason for the increased size of chitosan particles from higher air temperature might be due to a higher drying air temperature^36^, which may rapidly develop a dry, hard skin of chitosan droplet and prevent its subsequent deflation and shrinkage, resulting in a more massive particle. Figure 2 shows SEM micrographs of chitosan particles produced under different inlet air temperatures, with particles dried under higher inlet air temperatures (Fig. 2(c),(d)) showing a relatively smoother surface than those dried under lower temperatures (Fig. 2(a),(b)). However, the optimized inlet air temperature should not exceed 130 °C (Fig. 2(d)) in our case, with defects observed on top of the chitosan particles when using inlet air temperature of 150 °C (i.e., Fig. 2(e)), probably due to the internal vapor pressure exceeding the pressure that the external crust could withstand during faster drying process, in which case the semi-dried droplets might collapse and form defects much easier^10^.

Increasing feed flow rate might cause a collision and subsequent fusion of chitosan droplets leading to the increased particle size^36^. This observation is in agreement with our particle morphology results of SEM pictures shown in Fig. 3, with more of the bigger spherical particles observed when the air feed flow rate was increased (i.e., sample code from Q1 to Q5).

From these results, the particle size and morphology of spray-dried drug delivery system was found to be tunable by operating parameters of our spraying drying system, with the inlet air temperature lower than 130 °C and the feed flow rate of air of 0.075 L/min showing a better-controlled particle size and morphology of spray-dried chitosan particles, which would further benefit the fields of drug delivery and biopharmaceuticals. For example, particles with smaller scale such as 10 to 20 nm in diameter are usually more cytotoxic as opposed to larger ones (e.g., 50 to 100 nm in diameter), with particles <500 nm in diameter showing better performance than particles with diameter >500 nm in intravenous drug delivery due to their stronger circulate capability in blood which can target pathological tissues more specifically^37,38^.

In this study, TH was selected as a drug target model because of its widespread application in anti-inflammatory treatments with remarkable effects when the drug was delivered using polymeric carrier^20,23,39^. The release behavior of the TH from spray-dried chitosan-TPP particles was discussed by performing the dissolution test in phosphate buffer solution at a fixed pH of 7.4 to mimic the physiological values of intravenous (IV) administration^40^ or simulated intestinal fluid^41^ at constant precursor solution concentrations of TH and chitosan, 0.02 w/v% and 0.1 w/v%, respectively. TH releasing behaviors in buffers at a gastric fluid (pH 1.2) might be changed but not discussed here^42^.

Typically, the drug diffusion coefficient of a hydrogel decreases as the crosslinking density increases^43–45^ when the chitosan solution forms relatively stable matrix upon interaction with higher amount of TPP added, upon which the biopolymer chains form a diffusion barrier with smaller pore size^6,9,46^, making it difficult for the TH to pass through. For example, similar to drug molecules, the water uptake (swelling capacity) of the spray-dried chitosan microspheres was reported to decrease considerably with a higher amount of TPP added because more tightly cross-linked chitosan matrix does not swell (lower water uptake) as much as the loosely cross-linked chitosan matrix^47–49^. In this case, TPP content could alter the release of the drug from spray-dried TH/Chitosan-TPP particles, affording tunable drug release and pharmacokinetic profile of the loaded drug in our spray drying system.

However, it is essential to note that the stability of a colloidal system is related to the particle surface charges (i.e., zeta potential), with higher zeta potential favorable for stabilized particles due to a more significant electrostatic repulsion between particles to avoid the agglomeration^50^. The zeta potential of spray-dried chitosan-TPP microspheres were generally positively charged as indicated by the positive zeta potential reported^51^, with the charges mainly increased with increasing chitosan concentration or when the ratio of chitosan to TPP was improved, due to the reason that when the proportions of chitosan to TPP (higher amounts of TPP added) was lower, the protonated amino groups within chitosan were neutralized via TPP anions^52–54^. Therefore, although a tunable drug release could be obtained by altering cross-linking ratios of the chitosan-TPP system, the amount of TPP added must not be too high to avoid the undesired agglomeration phenomena due to the neutralized zeta potential values. As reported, zeta potential values of spray-dried chitosan particle were around +26 mV^41,49^, with the addition of TPP at 0.1% (w/v) dropping the values to +21 mV^41^, from which the TPP content investigated in our study (Table 3, with the weight to volume percentage of TPP in chitosan precursor solution, was ranged from 0.006 to 0.04% (w/v) < 0.1% (w/v)) should not be a concern regarding the TPP induced zeta potential drops.

In addition to the observations that decreasing porosity and water content is associated with slowed drug release^55^, drug loading efficiency might also affect the release rate of the drug from chitosan-based particles^56^. At lower drug loading (i.e., B1 in our case with the fastest drug releasing rate) with less TPP content, large pore fractions may be formed rapidly within the particle, which might increase the water uptake and consequently release the drug more quickly. The other explanation might be due to the smaller particle size of lower drug-loaded particles (e.g., particle diameters of B1 are smaller than that of B2 and B3 shown in Table 3 and Fig. 4), with larger surface area exposed for the faster drug release process observed^57^. On the other hand, a higher amount of aggregated TPP on the external phase of the TH/Chitosan-TPP particles (e.g., B5 in our case) has a comparatively reduced area exposed to the dissolution media, thus slow down the corresponding drug release.

Finally, the *in situ* solid-state transitions of TH under the test could affect the performance of the dosage form as well, with the fact that drugs may vary from amorphous molecules to well-defined crystalline structures. In our case, TH can exist either as an anhydrate (C_7_H_8_N_4_O_2_) or as a monohydrate (C_7_H_8_N_4_O_2_·H_2_O) with the anhydrate and the monohydrate belonging to the orthorhombic and the monoclinic crystal systems, respectively^58^. When placed in a humid environment, anhydrous TH might transform into a monohydrate form, and the dehydration of the monohydrate form would yield back the crystalline anhydrate. The chitosan might be modified as the excipient of the drug for improved mechanical strength and drug stability and delivery performances^59^.

By considering the complexity of the spray drying process, quality by design approach in the manufacturing process was, therefore important and required to carefully optimize the spray drying technology for long term stability of solid drug form under the test^60^.

## Material and Methods

### Chitosan properties and concentration in precursor solutions

Chitosan (75–85% deacetylated chitin from shrimp shell, with viscosity <20 cP for 1 wt% acetic acid (25 °C, Brookfield)(lit.**)**), with molecular weights <50,000 Da based on viscosity) from Sigma-Aldrich (St. Louis, MO, USA) was dissolved in aqueous acetic acid solution (1% v/v) at 6 different weight to volume percentages(w/v): 5 × 10^−5^, 5 × 10^−4^, 5 × 10^−3^, 0.01, 0.05, and 0.1 (w/v%).

The viscosity of 1.0 wt% chitosan solution prepared in aqueous acetic acid solution (1% v/v) was measured using Brookfield DV-III Ultra Programmable viscometer. The setting of the cone-on-plate viscometer was at 25 °C and different shear rates (25 to 250 (1/s)).

### TH-Chitosan-TPP ratios in precursor solutions

TH was purchased from Acros Organics USA (Morris Plains, NJ, USA). It was added into 0.1 w/v% chitosan precursor solutions under constant stirring (200 rpm) until homogenization at 0.02% (w/v). Sodium tripolyphosphate (TPP) from Sigma-Aldrich (St. Louis, MO, USA) was then added with five different concentrations at 0.006, 0.008, 0.01, 0.02, and 0.04 (w/v %) concerning the chitosan-TPP solution under gentle stirring (200 rpm). The TH-Chitosan-TPP precursor solution was then spray dried to obtain the Chitosan-TPP particles loaded with TH.

### Spray-drying operating conditions

**Figure 7:**
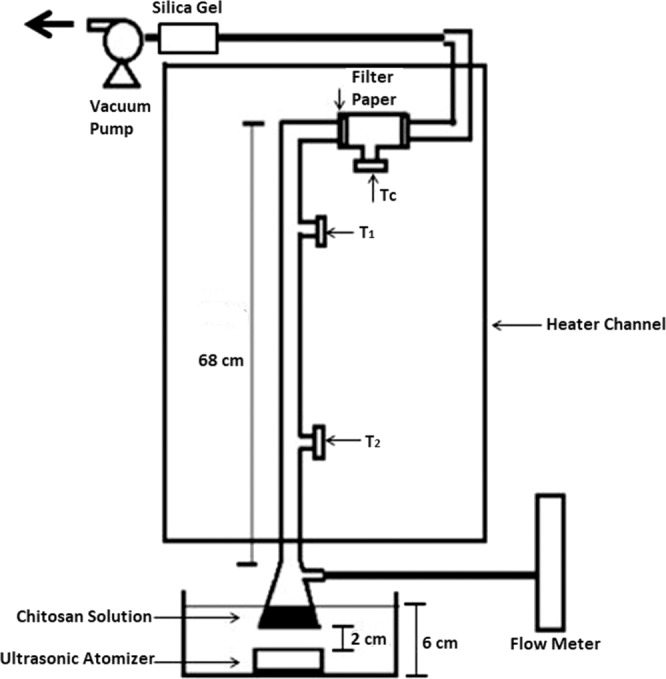
Spray drying apparatus. The drops were produced in ultrasonic atomization from a precursor solution (i.e., formulated solution from sections 2.1 and 2.2) and then vacuumed pumped into the central channel at a constant airflow rate (measured from the flow meter). The drier was heated with a heater strip wrapping around the column, of which the temperature was controlled by a temperature controller with an indicator (TC) and monitored at T1 and T2 position. The atomized drops carried by an airflow were heated and dried when it passed through the heated column, then collected the dried particles on a silicon plate and a filter paper (5 C) from Advantec MFS, Inc (Dublin, CA, USA).

A home-made spray drier was set up for drying the particles from precursor solutions, as shown in Fig. 7. The operating conditions are full factorial design, as demonstrated in Table 4. Primary sample codes are categorized by C, Q, T, and B for the representation of the amount of chitosan, the flow rate of air, the inlet temperature (Tc), and the amount of TPP respectively. The levels of primary experimental factors number secondary sample codes.

**Table 4:** The spray-dried particles prepared under various operating conditions.

Sample code	Chitosan %(w/v)	TH % (w/v)	TPP % (w/v)	Air Flow (L/min.)	Tc (°C)
C1	5 × 10^−5^	0	0	0.075	130
C2	5 × 10^−4^				
C3	5 × 10^−3^				
C4	0.01				
C5	0.05				
C6	0.1				
Q1	0.1	0	0	0.075	130
Q2				0.378	
Q3				0.681	
Q4				0.987	
Q5				1.589	
T1	0.1	0	0	0.075	70
T2					90
T3					110
T4					130
T5					150
B1	0.1	0.02	0.006	0.075	70
B2			0.008		
B3			0.01		
B4			0.02		
B5			0.04		

### Morphological identification

The shape and the surface of the spray-dried particles were observed under scanning electron nanoscopy (SEM, HITACHI S-3000H, Japan) at 5 kV accelerating voltage. The samples were previously sputter coated with Au/Pd using a vacuum evaporator (Balzers SDC 004 Sputter coater, Oerlikon Corporate Pfaffikon, Altendorf, Switzerland).

### Particle size and size distribution

SEM recorded a set of 15 images of chitosan particles with three representative graphs selected for counting the particles to determine the particle size distribution with a minimum of 300 particles for each sample selected using SetupWinB402 software, from which the mean particle diameter (D_n_) could be calculated according to Eq. (1).

1 $${D}_{n}=\sum ({n}_{i}\times {d}_{i})/\sum {n}_{i}$$

The particle size distribution was then described in a histogram showing a set of particle diameter (d_i_) specified on the horizontal axis and counting results (n_i_) of each diameter as the vertical axis. The geometric mean (d_50_) is the particle diameter that is equivalent to the 50% probability point, which indicates that half of the particulate mass is composed of particles more significant than this value, and half of the weight is composed of particles with smaller diameters. The diversity of the particle size (σ_g_) is described by the geometric standard deviation (G.S.D)^26^, determined from dividing the geometric mean by the particle size at the 84.13 percent probability (d_84.13_) by the geometric mean size (d_50_) (Eq. (2)).

2 $${\sigma }_{g}={d}_{84.13}/{d}_{50}$$

### Loading content

To determine the loading content of the TH/Chitosan-TPP particles drug delivery system, a known amount of spray-dried particles from fixed precursor solution concentration of TH (0.02 w/v%) and chitosan (0.1 w/v%) was left in a potassium phosphate buffer saline from Fisher Scientific (1x PBS, pH 7.4, Asheville, NC, USA) at 37 °C for 8 hours until total dissolution of TH from TH/Chitosan-TPP particles. The measurement was determined using a UV/Vis spectrophotometry (Ocean Optics TP300-UV/VIS) with the dip probe to have real-time monitoring at 270.04 nm. A calibration curve (i.e., y = 0.03405x − 0.02988, with y = absorbance and x = solution concentration of TH in PBS (mg/L), R^2^ = 0.995) was prepared by dissolving TH in 1x PBS in an appropriate range of concentrations (i.e., 3 × 10^−3^ mg/ml to 3 × 10^−2^ mg/ml in our case). Loading contents of TH from our TH/Chitosan-TPP particle system at various TPP contents were determined using Eq. (3).

3 $$loading\,content\,( \% )=\frac{TH\,detected\,after\,total\,dissolution\,from\,fixed\,numbers\,of\,microcapsules}{TH\,added\,to\,precursor\,solution\,for\,fixed\,numbers\,of\,microcapsules}$$

### *In Vitro* release profile of TH

The dissolution profile of TH was performed with 3 mg of spray-dried particles suspended in a release medium of 50 ml (1x PBS, pH 7.4). The amount of released TH was determined by spectrophotometrically (Ocean Optics TP300-UV/VIS) with the dip probe to have a real-time monitoring absorbance change at 270.04 nm, with the absorbance results correlated by drug concentration through a previously determined calibration curve (i.e., section 2.6.). All experiments were performed three times for two independent studies (n = 6), and the cumulative percentage of released TH was plotted versus time.

### Statistical analysis

All experiments were carried out in triplicate and repeated from two independent batch studies. Values were expressed as mean ± standard deviation (SD) and analyzed using Microsoft Excel (Redmond, WA, USA) software. The data were analyzed using a two-way analysis of variance (ANOVA) with the Tukey test for multiple comparisons. The significant differences were determined at P-value < 0.05 (n = 6)^61^.

## Conclusions

This study demonstrated that spray drying was the promising alternative method for TH encapsulation in chitosan particles with TPP as the crosslinker. The controllable particle size and sustained release of TH were determined by the selected process parameters and properties of the liquid feed of the spray drying system. The influence of operating conditions of the spray drying process was investigated on the size and morphology of spray-dried TH/Chitosan-TPP particles. As an increase in the inlet temperature of the drying air and feed air rate, the particle size increases as well. The established operating parameters lead to a smoother chitosan particle morphology. In particular, when the TH/Chitosan-TPP particles were prepared using a higher precursor concentration of chitosan at a selected carrying air flow rate and controlled drying temperature, the drug release rate was mainly tunable by the chitosan-TPP matrix density, which may lead to a better usage of TH/Chitosan-TPP particles in therapy for a more effective anti-inflammatory treatment.
